# The role of genes domesticated from LTR retrotransposons and retroviruses in mammals

**DOI:** 10.3389/fmicb.2012.00262

**Published:** 2012-07-27

**Authors:** Tomoko Kaneko-Ishino, Fumitoshi Ishino

**Affiliations:** ^1^School of Health Sciences, Tokai University,Isehara, Kanagawa, Japan; ^2^Medical Research Institute, Tokyo Medical and Dental University,Tokyo, Japan

**Keywords:** domesticated genes, LTR retrotransposons and ERVs, mammals, development and evolution

## Abstract

The acquisition of multiple genes from long terminal repeat (LTR) retrotransposons occurred in mammals. Genes belonging to a sushi-ichi-related retrotransposon homologs (*SIRH*) family emerged around the time of the establishment of two viviparous mammalian groups, marsupials and eutherians. These genes encode proteins that are homologous to a retrotransposon Gag capsid protein and sometimes also have a Pol-like region. We previously demonstrated that *PEG10* (*SIRH1*) and *PEG11/RTL1* (*SIRH2*) play essential but different roles in placental development. *PEG10* is conserved in both the marsupials and the eutherians, while *PEG11/RTL1* is a eutherian-specific gene, suggesting that these two domesticated genes were deeply involved in the evolution of mammals via the establishment of the viviparous reproduction system. In this review, we introduce the roles of *PEG10* and *PEG11/RTL1* in mammalian development and evolution, and summarize the other genes domesticated from LTR retrotransposons and endogenous retroviruses (ERVs) in mammals. We also point out the importance of DNA methylation in inactivating and neutralizing the integrated retrotransposons and ERVs in the process of domestication.

## INTRODUCTION

Domestication (exaptation, co-option) is an extended mode of restricting the retrotransposons, endogenous retroviruses (ERVs), and DNA transposons that are integrated into host genomes. It has been proposed that host organisms make use of such transposable DNA elements as a genetic resource of genes for novel purposes (Brosius and Gould, 1992; Smit, 1999). Telomerase, which maintains the telomere end repeats in chromosomes in eukaryotes, and two *recombination activating genes* (*RAG1* and *RAG2*) that are essential for producing the vast diversity of immunoglobulin types by V(D)J recombination in vertebrates, are good examples. The former was derived from a reverse transcriptase of a long terminal repeat (LTR) retrotransposon or retrovirus (Nakamura and Cech, 1998) and the latter from a transposase of a DNA transposon (Agrawal et al., 1998; Hiom et al., 1998). Mammalian centromere-associated protein B (CENP-B) facilitates centromere formation and is a DNA-binding protein derived from a transposase of the *pogo*-like DNA transposon family (Tudor et al., 1992; Casola et al., 2008). Although it bears considerable similarity to three fission yeast proteins, ARS-binding protein (Abp1), CENP-B homologs 1 and 2 (Cbh1 and Cbh2), which also exhibit centromere binding, it was recently reported that the origin of mammalian CENP-B is different from that of the three fission yeast proteins. That is, they are all derived from distinct *pogo*-like DNA transposons, indicating that convergent domestication occurred in the mammalian and fission yeast lineages. In plants, the Arabidopsis *far-red elongated hypocotyls 3* (*FHY3*) and *far-red-impaired response* (*FAR1*) genes are derived from an ancient Mutator-like transposase, a kind of DNA transposons. They encode transcription factors essential for the light response via phytochrome A signaling (Lisch et al., 2001; Hudson et al., 2003; Lin et al., 2007). From these data, it is clear that the domestication of transposable elements had a profound effect on quite a large numbers of animals and plants during the course of biological evolution, even though only few cases are currently known.

The retrotransposons, ERVs, and their remnant DNA sequences occupy approximately 40% of the mammalian genome and they have long been thought to be either “selfish” genes or useless “junk.” Is it thus the case that domestication events are very rare in mammals? Are there any domesticated genes which are present in a mammalian-, therian-, and eutherian-specific manner? Alternatively, are there domesticated genes which have been conserved in a more restricted manner, i.e., as species- and strain-specific genes? If so, it would be highly probable that they have contributed to mammalian evolution in various ways and to different degrees.

**FIGURE 1:**
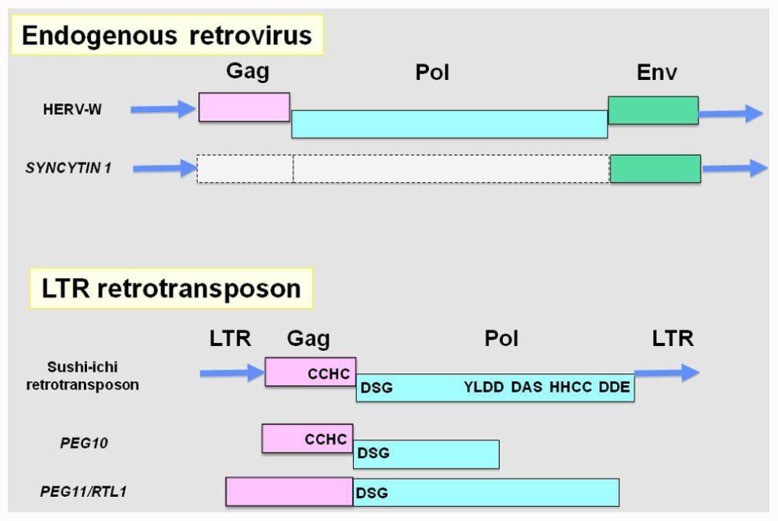
**Endogenous retrovirus, LTR retrotransposon and their domesticated genes**. *Top*: an endogenous retrovirus, HERV-W, and *SYNCYTIN 1*. *SYNCYTIN 1* retains LTRs at both ends and the *Env* gene, while the *Gag* and Pol genes do not support ORFs corresponding functional proteins because of stop mutations. *Bottom*: an LTR retrotransposon, sushi-ichi, and the domesticated *PEG10* and *PEG11/RTL1* genes. Both *PEG10* and *PEG11/RTL1* have lost LTRs while entire regions exhibit significant homologies to the Gag and Pol genes remaining in some of the retrotransposon domains. The -1 frameshift mechanism is conserved in *PEG10*. LTR, long terminal repeat; Gag, group-specific antigen; Pol, polymerase; Env, envelope; CCHC, RNA-binding motif; DSG, protease active site; YYDD, reverse transcriptase; DAS, RNase highly conserved motif; HHCC, integrase DNA binding motif; DDE, strongly conserved integrase.

The recent availability of mammalian genome sequence information enabled us to identify dozens of novel domesticated genes from LTR retrotransposons/ERVs. In 2000, human *SYNCYTIN* (*ERVWE1*) was identified as the first candidate domesticated gene derived from ERVs in mammals (Blond et al., 2000; Mi et al., 2000). As shown in **Figure 1**, it derives from an *envelope* (*Env*) gene of a human-specific endogenous retrovirus, HERV-W, and was suggested by *in vitro* study to mediate placental cytotrophoblast fusion so as to produce syncytiotrophoblast cells in human placental morphogenesis. Interestingly, humans have two *SYNCYTIN* genes, but they are primate-specific genes (Blaise et al., 2003). Similar genes (also called *Syncytin*) were also discovered in several mammalian lineages that were independently acquired from *Env* genes from different ERVs (Dupressoir et al., 2005; Heidmann et al., 2009). Finally, mouse *SyncytinA* and *B* have been to be essential placental genes using knockout mice (Dupressoir et al., 2009, 2011).

In 2001, the first and second candidate domesticated genes from a sushi-ichi-related LTR retrotransposon were identified as *paternally expressed 10* (*PEG10*; Ono et al., 2001) and *paternally expressed 11/retrotransposon-like 1* (*PEG11/RTL1*; Charlier et al., 2001). They encode proteins homologous to a retrotransposon Gag and a Pol protein, respectively (**Figure 1**). Combined with definitive genetic studies using knockout mice, *PEG10* and *PEG11/RTL1* have been shown to be essential for mammalian development via placenta formation and the subsequent maintenance of its placental function, respectively (Ono et al., 2006; Sekita et al., 2008). As *PEG10* is conserved in all the eutherian and marsupial species, it is a therian-specific gene (Suzuki et al., 2007), while *PEG11/RTL1* is eutherian-specific (Edwards et al., 2008). All these findings demonstrated that these two domesticated genes are essential in the current mammalian developmental system and indicate that they have been critically involved in the establishment and diversification of viviparous mammals. In other words, these domesticated genes could be major players in the macroevolution of mammals (Kaneko-Ishino and Ishino, 2010).

The concept of macroevolution by such domesticated genes from the LTR retrotransposons/ERVs and the DNA transposons, as well as rewiring gene regulatory networks by non-LTR retrotransposons (Kuwabara et al., 2009; Lynch et al., 2011; Schmidt et al., 2012) is a subject of interest not only to biologists, but also to those in the general public who are interested in biological evolution and the origin of human beings. It is of special interest because it implies the existence of a unique long-term relationship between the transposable elements and the emergence of mammals.

In this review, we introduce the essential role played by *PEG10* and *PEG11/RTL1* in mammalian development via placenta formation, and summarize the current understanding of domesticated genes from the LTR retrotransposons/ERVs, especially those in the mammalian lineages. We also discuss the critically important role of DNA methylation in the process of retrotransposon domestication.

## *PEG10* AND *PEG11/RTL1* IN MAMMALIAN DEVELOPMENT AND EVOLUTION

**FIGURE 2:**
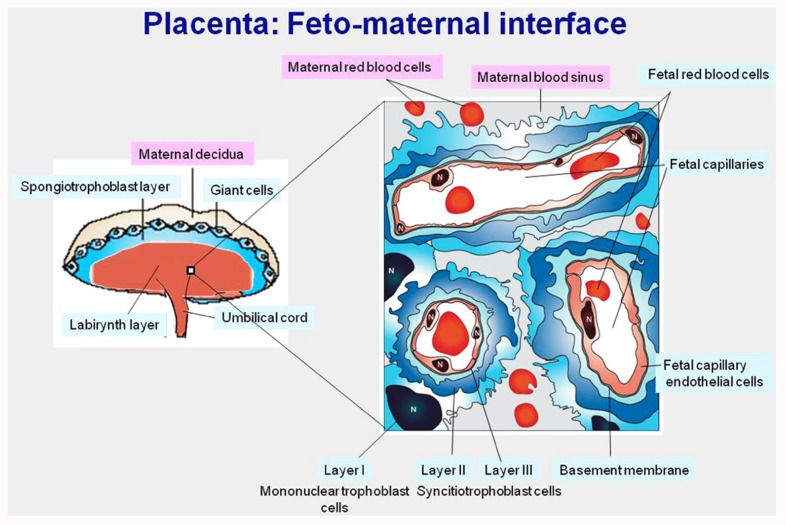
**Mouse mature placenta**. *Left*: mouse placenta is composed of labyrinth, spongiotrophoblast, and giant cell layers. *Right*: magnified view of the labyrinth layer. In the labyrinth layers, fetal capillaries are surrounded by three layers of trophoblast cells and are bathed in maternal blood, functioning as a site of nutrient and gas exchange between the fetal and maternal blood.

*PEG10* and *PEG11/RTL1* were identified as paternally expressed genes in the course of an investigation on genomic imprinting (Charlier et al., 2001; Ono et al., 2001). Genomic imprinting is a mammalian-specific epigenetic mechanism regulating the parent-of-origin expression of a subset of specific genes. For these imprinted genes, the two parental alleles are not equivalent: some of the genes are transcribed only from maternally transmitted alleles (maternally expressed genes, *MEGs*) and the others are transcribed only from paternally transmitted alleles (paternally expressed genes, *PEGs*; Kaneko-Ishino et al., 2006). Then, genomic imprinting plays an essential role in mammalian development, growth, and behavior via the activity of certain critically important imprinted genes. In mice, there are more than 10 imprinted regions which have been identified, consisting of both *PEGs* and *MEGs*. Among them, a proximal region of chromosome 6 is known to cause early embryonic lethality upon maternal duplication, while maternal duplication of a distal region of chromosome 12 causes late embryonic/neonatal lethality associated with growth retardation (Cattanach and Beechey, 1990; see also Genomic imprinting map: http://www.har.mgu.ac.uk/research/genomic_imprinting/). Mouse *Peg10* and *Peg11/Rtl1* are the major genes responsible for the lethal phenotypes observed in these imprinted regions, respectively (Ono et al., 2006; Sekita et al., 2008). Using knockout mice, we demonstrated that *Peg10* and *Peg11/Rtl1* play essential roles in early placenta formation and maintenance of the placenta in the mid-to-late stages of gestation, respectively. No labyrinth or spongiotrophoblast formation was observed in the placenta of *Peg10* knockout mice. The labyrinth layer is a central part of the mouse placenta in which feto-maternal interactions take place. A large portion of the fetal capillaries exist in the labyrinth layer and allow an exchange of nutrients and gases between maternal and fetal blood cells (**Figure 2**). Mouse embryos require nutrient supply from the placenta starting on day 9.5 of gestation, therefore, *Peg10* KO embryos do not survive beyond this stage.

*PEG10* encodes two open reading frames exhibiting the highest homology to the Gag and Pol proteins of the sushi-ichi retrotransposon, respectively, and produce two types of proteins, one derived from ORF1 and the other from both ORF1 and 2 (Ono et al., 2001; Volff et al., 2001; **Figure 1**). The PEG10 protein retains a CCHC RNA-binding motif in the Gag protein and there is a DSG protease domain in the Pol protein. The -1 frameshift mechanism which produces a Gag–Pol fusion protein that is unique to LTR retrotransposons and ERVs is conserved in *PEG10*, providing strong evidence for its origin from an LTR retrotransposon (Ono et al., 2001; Shigemoto et al., 2001; Manktelow et al., 2005). The biochemical function of the PEG10 protein has yet to be elucidated. However, it was reported that *PEG10* is highly expressed in a great majority of hepatocellular carcinomas and confers oncogenic activity. Furthermore, the PEG10 protein is reportedly associated with a member of the “seven in absentia homolog” family (SIAH1 protein) that acts as a mediator of apoptosis. Overexpression of PEG10 decreased the cell death mediated by SIAH1, suggesting that PEG10 has a growth promoting function related to apoptosis in somatic cells (Okabe et al., 2003).

**FIGURE 3:**
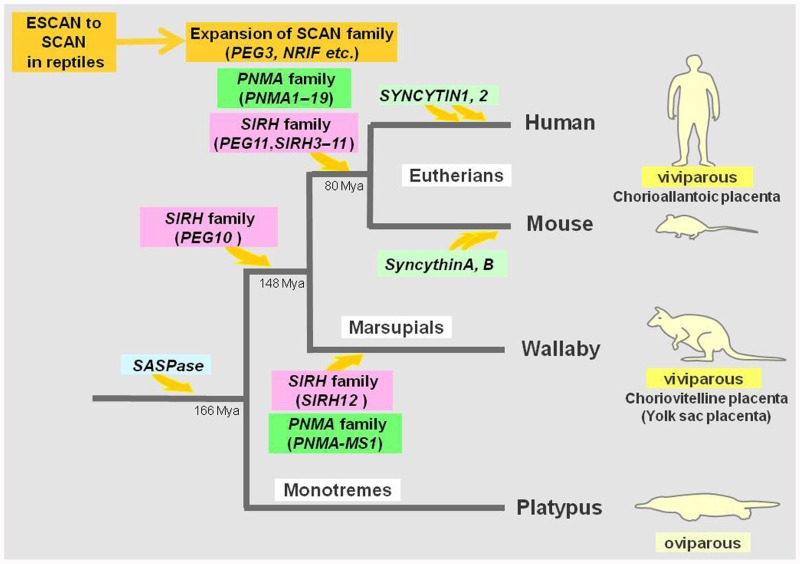
**Domestication from LTR retrotransposons and ERVs in mammals**. The acquisition of *SASPase* occurred in a common mammalian ancestor. *PEG10* was domesticated in a common therian ancestor while *PEG11/RTL1*, *SIRH3–11*, and *PNMA1–19* were domesticated in a common eutherian ancestor with subsequent loss of some of the *PNMA* genes in rodents. The ESCAN domain was domesticated in lower vertebrates and its transition to the SCAN domain took place by combining with the zinc finger and/or KRAB motifs which had already occurred in certain reptiles. In any event, the expansion of SCAN family is obvious in the eutherians. *SIRH12* and *PNMA-MS1* are derived from marsupialspecific domestication events. The *SYNCYTINs* were independently recruited in several different mammalian lineages. The eutherians and marsupials are viviparous, having chorioallantoic and choriovitelline placentas (yolk sac placentas), respectively. Both *PEG10* and *PEG11/RTL1* are essential for the proper formation of efficient chorioallantoic placentas.

The genomic record shows that *PEG10* is conserved in the eutherian and marsupial mammals among the vertebrates (Suzuki et al., 2007). As the placenta is an organ unique to the viviparous reproduction system in these two mammalian groups, it is clearly evident that this gene domesticated from the LTR retrotransposon contributed to the establishment of the current developmental systems of viviparous mammalian groups as a positively selected gene (Suzuki et al., 2007; Kaneko-Ishino and Ishino, 2010). Thus, *PEG10* is a very good example of Darwinian evolution and natural selection at work in a macroevolutionary process beyond the individual species which led to the establishment of a subclass of mammals, the therians (**Figure 3**).

The PEG11/RTL1 protein also possesses homology to both the Gag and Pol proteins, including the DSG protease domain in the latter, although no frameshift is required in this case (Charlier et al., 2001). The amino acid sequence homology between the PEG10 and PEG11/RTL1 proteins is approximately 20–30%, indicating their different functions. Mouse *Peg11/Rtl1* knockout clearly showed that *Peg11/Rtl1* has both a different role from *Peg10* and is essential for the maintenance of placental function in the mid-to-late fetal stages (Sekita et al., 2008). *Peg11/Rtl1* is expressed in endothelial cells (of extraembryonic mesoderm lineage) of the fetal capillaries in the labyrinth layer, in contrast with *Peg10*, which is expressed in the labyrinth and spongiotrophoblast cells (of extraembryonic endoderm lineage; **Figure 2**). As mentioned above, the fetal capillary is the place where feto-maternal interaction occurs. The loss of *Peg11/Rtl1* causes clogging in many of the fetal capillaries in the labyrinth layer because of the phagocytosis of endothelial cells carried out by the surrounding trophoblast cells. The Peg11/Rtl1 protein may protect endothelial cells against placental trophoblast cells, which have a highly invasive and hence dangerous nature, although its biochemical function awaits demonstration. It should be noted that the loss and overexpression of *PEG11/RTL1* are thought to attribute to the etiology of two different human imprinted diseases, maternal and paternal disomies of human chromosome 14 (matUPD14 and patUPD14), where *PEG11/RTL1* is located, respectively (Kagami et al., 2008). In these cases, *PEG11/RTL1* plays a major role, not only in placental function, but also in fetal and postnatal growth.

*PEG11/RTL1* is conserved in all eutherian mammals but is absent from marsupial mammals, and is, therefore, a eutherian-specific gene (Edwards et al., 2008). Marsupials use a choriovitelline placenta (yolk sac placenta), which is different from the eutherian chorioallantoic placenta and give birth to their young after a very short gestation period compared to the eutherians (Renfree, 2010). PEG11/RTL1 function is necessary for the latter to complete their longer gestational period. Therefore, it is probable that *PEG11/RTL1* has a role in the reproduction system of eutherians, which have the chorioallantoic placenta and that it thus contributed to the diversification of these two viviparous mammalian groups. We can say that *PEG11/RTL1* provides another good example of macroevolution in mammals (i.e., the establishment of an infraclass of mammals, the eutherians) by domesticated genes from LTR retrotransposons (Kaneko-Ishino and Ishino, 2010; **Figure 3**).

## OTHER *SIRH* FAMILY GENES DERIVING FROM THE SUSHI-ICHI-RELATED RETROTRANSPOSON

*PEG10* and *PEG11/RTL1* belong to a sushi-ichi-related retrotransposon homolog (*SIRH*) family consisting of 12 genes, including these two genes as *SIRH1* and *SIRH2*, respectively (**Figure 2**; Ono et al., 2006). It is also called the mammalian-specific retrotransposon transcripts (*MART*; Brandt et al., 2005a,b) or *SUSHI* family (Youngson et al., 2005). The *SIRH1–11* genes are conserved in the eutherian species but no marsupial orthologs have been found, yet nevertheless, *SIRH12* is derived from a marsupial-specific insertion event (Ono et al., 2011; **Figure 3**). Among the *SIRH* family genes, *PEG10* (*SIRH1*), *PEG11/RTL1* (*SIRH2*), and *SIRH9* share homology to both the Gag and Pol proteins, while all the others bear homology only to the Gag protein, but encode proteins of more than 100 amino acid sequences (Brandt et al., 2005a,b; Youngson et al., 2005; Campillos et al., 2006; Ono et al., 2006).

*SIRH12* is present in the tammar wallaby, an Australian marsupial species, but its amino acid sequence is degenerated in the gray short-tailed opossum, a South American marsupial species, suggesting that it is only functional in the former (Ono et al., 2011). No ortholog has been reported in any eutherian species in the corresponding region between the *ectodermal-neural cortex* (*ENC1*) and *rho-guanine nucleotide exchange factor* (*RGNEF*) genes where wallaby *SIRH12* and opossum pseudo *SIRH12* are located in the marsupial genome. These findings demonstrate that the *SIRH* family of genes appeared mainly around the establishment of the therian mammals, one (*PEG10*) before and all the others after the split of the marsupials and eutherians (**Figure 3**). Consequently, the eutherians and the marsupials have a different set of *SIRH* family genes except for *PEG10*. It is likely that *SIRH3–11* and *SIRH12* also have roles in the eutherian and marsupial developmental and reproductive systems as well as *PEG10* and *PEG11*. As they are expressed in the brain, ovary, and testis as well as the placenta, they may be related to ovulation, gestation, delivery, and/or maternal nursing behaviors, including lactation, as well as placenta formation. Their respective functions are now under investigation using knockout mice.

## *PNMA*-FAMILY GENES FROM THE Gypsy12_DR-RELATED LTR RETROTRANSPOSON

A paraneoplastic Ma antigen (*PNMA*) family is another large family consisting of eutherian- and marsupial-specific genes (Schüller, 2005; Iwasaki et al., in preparation; **Figure 3**). *PNMA*-family genes (*PNMA1–3*) were first identified as genes encoding neuronal auto-antigens using sera from human patients with paraneoplastic neurological syndromes (Voltz et al., 1999; Rosenfeld et al., 2001). By comprehensive search of a protein database, Schüller (2005) identified additional human *PNMA* genes, *MOAP1/PNMA4*, *PNMA5*, and *PNMA6*, among which *PNMA6* has no mouse ortholog. Campillos et al. (2006) performed a genome-wide search for *PNMA* genes and identified a total 15 genes and 1 pseudogene in humans. They also showed that all of the *PNMA* genes were related to a Gypsy12_DR-related Gag protein group of the Ty3/Gypsy LTR retrotransposons isolated from zebrafish (*Danio rerio*) and that there was no Gypsy12_DR-derived sequences in birds. Recently, Iwasaki and colleagues identified novel *PNMA* genes by a re-examination of the entire mouse and human entire genome sequences and the *PNMA*-family genes found thus far number 15 and 19 in mice and humans, respectively; all of these genes have Gag-like proteins, but none are homologous to the Pol protein. The difference in number may be due to the rodent-specific deletion of the *PNMA6A–6D* genes on X chromosome (Schüller, 2005; Iwasaki et al., in preparation).

No knockout mouse studies on *PNMA* genes have been reported, but there are reports indicating that these genes are involved in important biological pathways and related to human diseases. *PNMA4*-deficient cells exhibit aggressive anchorage-independent growth, suggesting that *PNMA4* has an important role in regulating apoptosis signaling in a strict temporal manner in mammalian cells, because the PNMA4/MOAP-1 protein is short-lived and constitutively degraded by the ubiquitin–proteasome system (Lee et al., 2009).

Cho et al. (2008a), 2011 reported *PNMA10* to be a candidate gene for X-linked mental retardation (*XLMR*) in humans. In mice, *Pnma10/Zcchc12* is expressed in the embryonic ventral forebrain in a cholinergic–neuron-specific manner (Cho et al., 2011), and is known to act as a transcriptional co-activator for bone morphogenic protein (BMP) signaling by binding to the SMAD family of proteins (Cho et al., 2008b). Therefore, it is likely that *PNMA10* is related to the evolution of brain function in mammals.

Recently, *Pnma14/CCDC8* was suggested to be one of the genes responsible for 3-M syndrome (Hanson et al., 2011a,b). 3-M syndrome is an autosomal-recessive disease characterized by severe postnatal growth restriction, leading to a significantly diminished stature. *CULLIN7* (*CUL7*) **and *Obsculin-like 1* (*OBSL1*) are both related to the transcription of *insulin-like growth factor-binding protein* (*IGFBP*) genes and have been identified as two of the genes involved in 3-M syndrome (Huber et al., 2005; Hanson et al., 2009). Importantly, the OBSL1 protein interacts with both the PNMA14/CCDC8 and CUL7 proteins, indicating that this protein complex is responsible for the growth retardation observed in 3-M patients. These findings suggest that *PNMA* genes play an important role in the eutherian development and growth that are impacted by human diseases.

We have recently identified two marsupial-specific *PNMA* genes, *PNMA-MS1* and *-MS2* (Iwasaki et al., in preparation; **Figure 3**). *PNMA-MS1* exists in the same genome location in both the Australian (tammar wallaby) and South American (gray short-tailed opossum) marsupial species, but no orthologs exist in the eutherians, suggesting that they are derived from a marsupial-specific domestication event similar to that of *SIRH12*. *PNMA-MS2* was only found in the opossum because there is a gap in the corresponding region of the wallaby genome sequence. However, it is clear that no ortholog exists in any eutherian species. Thus, it is also clear that *PNMA*-family genes were independently domesticated in the eutherian and the marsupial lineages, and may have certain eutherian- and marsupial-specific functions, respectively.

## THE RETROVIRAL-LIKE ASPARTIC PROTEASE SASPase IS CONSERVED IN MAMMALS

Skin aspartic protease (SASPase), which is known a retroviral-like aspartic protease (Bernard et al., 2005), plays a key role in determining the texture of skin by modulating the degree of hydration via the processing of profilaggrin (Matsui et al., 2006, 2011; Barker et al., 2007). *SASPase* is a single gene conserved in the eutherians, marsupials, and presumably the monotremes (Matsui, personal communication), and thus is a mammalian-specific gene (**Figure 3**). The profilaggrin protein comprises a tandem array of filaggrin monomers and the SASPase is its specific protease which produces the filaggrin monomer (Matsui et al., 2011). The *Filaggrin* gene has recently been identified to be etiologically responsible for atopic dermatitis (Barker et al., 2007). Interestingly, both SASPase and profilaggrin are unique to mammals and expressed exclusively in the stratified epithelia in skin. Therefore, it is highly likely that they contributed to the establishment of the mammalian-specific skin barrier system. Aberrant *SASPase* expression in transgenic mice reportedly leads to impaired skin regeneration and skin remodeling after cutaneous injury or chemically induced hyperplasia (Hildenbrand et al., 2010), and *SASPase*-deficient mice exhibit fine wrinkles on the sides of the adult body (Matsui et al., 2006).

## SCAN-FAMILY GENES RAPIDLY EXPANDED IN THE COURSE OF EUTHERIAN EVOLUTION

The SCAN-family is not a mammalian-specific gene family because its ancestral form exists in non-mammalian vertebrates, but nevertheless, an enormous expansion occurred in the eutherian species (**Figure 3**). The SCAN motif consists of only a C-terminal portion of the Gag capsid (CA) protein and, in mammals, it always accompanied by multiple C2H2 zinc finger motifs and/or Krüppel-associated box (KRAB) domains neither of which is of retrotransposon origin. It is suggested that the former part was already domesticated at or near the root of the tetrapod animal branch from a full-length CA gene derived from a Gmr1-like retrotransposon. This is called the extended SCAN (ESCAN) domain and that either it or its truncated SCAN motif combined with the zinc finger and/or KRAB motifs in the Anolis lizard (Emerson and Thomas, 2011). Approximately, 60 and 40 genes are known in humans and mice, respectively, and some of them are involved in development and differentiation as transcription factors, such as *ZNF202*, *ZNF197*, *ZNF444*, *ZNF274* (*neurotrophin receptor interacting factor*, *NRIF*), *Zfp496* (*NSD1-interacting zinc finger protein 1*, *Nizp1*) and *Zfp263* (*NT2*; Edelstein and Collins, 2005).

Therefore, it is highly likely that some of the *SCAN*-family genes are related to certain eutherian-specific functions. One example is *paternally expressed gene 3* (*PEG3*; Kuroiwa et al., 1996) that was reported to be essential for maternal nursing behavior as well as promoting embryonic growth (Li et al., 1999). The *PEG3* protein has very unique structural features among C2H2 zinc finger proteins, such as amino acid sequences for 11 C2H2 zinc finger motifs and a wider spacing of these motifs. The C2H2 zinc finger proteins comprise the largest class of eukaryotic transcription factors, yet no other C2H2 zinc finger proteins have such features (Kuroiwa et al., 1996). *PEG3* is widely expressed during fetal development of mice, and strongly in adult neurons and skeletal muscle. The *Peg3* KO offspring are approximately 20% smaller at birth, with markedly reduced nursing behavior and a reduced number of oxytocin-positive neurons in the hypothalamus of *Peg3* KO females (Li et al., 1999). Human *PEG3* has tumor-suppressing activity in glioma cell lines by its capacity to inhibit Wnt signaling, and the loss of its expression is reportedly observed in gliomas (Kohda et al., 2001; Maekawa et al., 2004; Jiang et al., 2010).

## INDEPENDENT DOMESTICATION EVENTS OF THE *SYNCYTINs* IN DIFFERENT LINEAGES IN EUTHERIANS

As mentioned in Section “Introduction,” *SYNCYTIN* was first discovered in humans (Blond et al., 2000; Mi et al., 2000). Although there are many *Env*-related DNA sequences in the human genome, only two exhibit fusogenic activity in cell fusion assays and now these are called *SYNCYTIN1* and *2* (Blaise et al., 2003). They are derived from different human-specific ERVs, HERV-W, and HERV-FRD, and became integrated into a primate lineage 25 and >40 MYA, respectively (**Figure 3**). Recent studies demonstrated that similar genes exist in an order- or family-specific manner in several mammalian lineages, i.e., producing syncytiotrophoblast cells by cell fusion in the placenta. Mice also have two *Syncytin* genes, *SyncytinA* and *B*, derived from Muridae family-specific integrations of HERV-F/H-related ERV(s) approximately 20 MYA (Dupressoir et al., 2005; **Figure 3**), and rabbits (*Oryctolagus cuniculus*) have another *SYNCYTIN-Ory1* from Leporidae family-specific integration of a different type-D retrovirus 12–30 MYA (Heidmann et al., 2009). Therefore, at least three independent domestication events have been confirmed in the eutherians, indicating that domestication from ERVs which were actively functioning during the time of mammalian radiation.

*SyncytinA* knockout mice exhibit mid-fetal lethality because of the structural abnormality of the placenta (Dupressoir et al., 2009), and double knockout of both *SyncytinA* and *B* causes an even more severe phenotype, early embryonic lethality (Dupressoir et al., 2011). Among the eutherians, placental morphology and functions are quite substantially diverged. Therefore, it is very interesting that the *SYNCYTINs* from the ERVs appear to have important roles in the placenta that they play in an order- or family-specific manner, while *PEG10* and *PEG11/RTL1* from the LTR retrotransposons are conserved in the therians and eutherians, respectively, and presumably have contributed to the establishment of the basic structure of viviparous reproductive systems in the current eutherian species.

## RESISTANCE TO VIRAL INFECTION BY DOMESTICATED VIRAL GENES

ERVs have long been thought to confer resistance to infection by exogenous retroviruses. Well-known examples are *Friend virus susceptibility 1* and *4* (*Fv1* and *Fv4*) and *resistance to mink cell focus-forming (MCF) virus* (*Rmcf*) genes, which exhibit resistance to murine leukemia viruses (MuLVs) in mice (Pincus et al., 1971; Suzuki, 1975; Hartley et al., 1983). *Fv1* is derived from the Gag region of an ancient MERV-L element (Best et al., 1996; Bénit et al., 1997), whereas *Fv4* and *Rmcf* consist of intact *Env* genes, the expression of which prevents infection via receptor interference (Ikeda et al., 1985; Lyu and Kozak, 1996; Taylor et al., 2001; Jung et al., 2002). Endogenous betaretroviruses (enJSRVs) in sheep are another example (Dunlap et al., 2005). The ovine genome possesses approximately 20 copies of enJSRVs that are highly related to two exogenous oncogenic viruses, Jaagsiekte sheep retrovirus (JSRV) and enzootic nasal tumor virus. It has been proposed that the enJSRVs *Env* genes are beneficial to the host and help protect of the uterus from viral infection and act as regulators of placental morphogenesis and function. They exist as species- or strain-specific genes, meaning that they are derived from recent domestication events compared to the *SIRH*-, *PNMA*-, and SCAN-family genes as well as the *SASPase* gene.

Therefore, it is clear that the domestication from LTR retrotransposons and ERVs has a very long history, dating from around the time of the establishment of vertebrates, on through the establishment and diversification of mammals and ultimately to the radiation of each mammalian species. Koala retrovirus (KoRV) has recently been reported to cause leukemia, lymphoma, and immunosuppression in the Australian Koala population (Tarlinton et al., 2006, 2008; Stoye, 2006). Interestingly, KoRV is currently undergoing endogenization and it is likely that it entered the koala genome within the last 200 years. Therefore, retrotransposon endogenization may be a fairly ordinary process in the long course of evolution, and novel genes may continue to appear by this mechanism in the future.

## GENE DUPLICATION OF DOMESTICATED GENES

Although dozens of domesticated genes have been found in mammals, this does not necessarily mean that independent domestication events have happened as often as the number of domesticated genes. Certain domesticated genes have apparently been produced by the gene duplication of an originally domesticated gene, such as in the SCAN family of genes. The SCAN domain was domesticated long before the emergence of mammals in the lower vertebrates (ESCAN) and then a new combination of this domain and zinc finger and/or KRAB motifs produced the SCAN-family gene prototype in a reptile, and its expansion occurred during radiation of the eutherians (Emerson and Thomas, 2011; **Figure 3**).

*SIRH4*, *5*, and *6* as well as *PNMA6A*, *6B*, *6C*, and *6D*, are other clear examples of gene duplication. The domestication of the original gene must have occurred in the ancestral eutherian mammals, but these clusters were produced by gene duplication because they encode very nearly the same coding frames. It is interesting to elucidate whether they are in the process of diversifying into genes with different functions or there is some as yet unknown reason for them to multiply and increase their copy numbers in this way. Nevertheless, as discussed above, at least two independent domestication events occurred in the eutherians and the marsupials in the *SIRH* and *PNMA* families, and at least four independent domestications have been confirmed in three different eutherian lineages in the case of the *SYNCYTINs* (**Figure 3**).

## THE ESSENTIAL ROLE OF DNA METHYLATION IN THE DOMESTICATION PROCESS

Retrotransposons are potentially harmful to host organisms because their integration not only causes genetic diseases by disrupting essential genes, but also induces chromosomal deletion as well as recombination by DNA homologous recombination between the two of them. Their integration could also disturb transcription of neighboring genes. Thus, host organisms must prevent any further propagation that would result in an accumulation of new insertion events by regulating their transcription. How are they critically silenced and yet stably inherited from generation to generation in a manner similar to endogenous DNA sequences in the host genome?

Mammals have adopted certain defense mechanisms against them, such as DNA methylation and histone modifications (Rowe and Trono, 2011). The integrated retrotransposons are usually heavily DNA methylated and transcriptionally silenced in almost all somatic cells. They have the character of being neutral genes in the mammalian genome. According to the neutral theory of molecular evolution proposed by Kimura (1968), 1983, such neutral mutations are fixed in a population by the mechanism of random drift. Ohta (2002) proposed in her “nearly neutral theory” as an extension of neutral evolution that less harmful mutations could become fixed in a population if the population size were sufficiently small). We previously proposed the hypothesis that in the course of retrotransposon domestication the neutral or nearly neutral evolution preceded Darwinian evolution and helped supply novel genes for novel purposes from the integrated retrotransposons (Kaneko-Ishino et al., 2006; Kaneko-Ishino and Ishino, 2010). In brief, we assume that either neutral or nearly neutral evolution played essential background roles by both inactivating and neutralizing integrated retrotransposons. Subsequently, their gradual conversion from silenced harmful genes to slightly advantageous genes took place as the result of multiple mutations. A loss of such silencing, at least in a subset of tissues, was ultimately required for the “new gene” to have a certain function. Darwinian forces then came into play, and by natural selection certain of these genes became more usefully functional and thus advantageous for the host organisms. It should be noted that in extraembryonic organs, such as the yolk sac and placenta in mammals, the DNA methylation levels are lower than those in other embryonic and adult tissues. Therefore, a leaky expression of retrotransposons and retroviruses constantly occurs. In this situation, the integrated retrotransposons and their subsequent mutated forms would be less harmful. However, in the case of advantageous mutations, a swift transition from the state of nearly neutral evolution to that of Darwinian evolution would take place. In this regard, the extraembryonic tissues might have been a site of retrotransposon domestication during the course of mammalian evolution, which is consistent with the fact that the domesticated *PEG10*, *PEG11/RTL1*, and *SYNCYTIN* genes play essential roles in the placenta (Kaneko-Ishino and Ishino, 2010).

In this hypothetical scenario, various epigenetic mechanisms, such as DNA methylation and/or histone modification, might have played a critical role. In mammals, DNMT1 is the essential maintenance DNA methyltransferase and the loss of its activity causes early embryonic lethality associated with overexpression of IAP retrovirus (Li et al., 1992; Walsh et al., 1998). The two *de novo* DNA methyltransferase DNMT3A and DNMT3B are also essential for mammalian development, and the loss of their activities causes lethality in the postnatal and embryonic period in mice, respectively (Okano et al., 1999). Overexpression of IAP retrovirus was also observed in *Dnmt3a* and *3b* double knockout mice, although to a lesser degree than *Dnmt1* KO mice (Okano et al., 1999). DNMT3L does not have DNA methyltransferase activity itself, but has an essential function of producing a different DNA methylation status in female- and male-derived genomic DNA in the process of establishing the genomic imprinting memories associated with *DNMT3A* (Bourc’his et al., 2001; Hata et al., 2002). It is known that this complex is also essential for retrotransposon methylation in the paternal germ line (Bourc’his et al., 2001; Bourc’his and Bestor, 2004). The coincident emergence of *DNMT3L* in the therian mammals is highly suggestive, both for the origin of the genomic imprinting mechanism as well as the abundance of LTR retrotransposons/ERVs, each of which is specific to the therian genome (Yokomine et al., 2006). It should be noted that H3K9 methyltransferase ERG-associated protein with SET domain (ESET, also called SETDB1) coupled with KRAB-associated protein 1 (KAP1, also called TRIM28) and zinc finger protein ZFP806 is required for H3K9 trimethylation as well as the repression of the retrotransposons and ERVs in undifferentiated mouse ES cells (Wolf and Goff, 2007, 2009; Matsui et al., 2010). Such a DNA methylation-independent pathway may be necessary, because DNA methylation is dynamically reprogrammed during the early embryonic period in mammals.

Finally, we would like to consider how the mammalian viviparous reproductive system originally started using the retrotransposon-derived *PEG10* gene. If this new reproductive system first happened in a single individual, was it possible for such an individual to survive and propagate his or her offspring? It is worth mentioning that the nearly neutral theory of molecular evolution can also explain how new species originated not from a single individual, but rather from a population subset (Kimura, 1983). Preadaptive mutations were already distributed in a neutral manner. Adaptive functions emerged under the selective pressures of a new environment. This suggests the neutral evolution process could also play a role as an “evolutionary capacitor,” as predicted in the case of heat shock protein (Hsp) 90, where genotypic variations in other genes are masked and therefore are accumulated without causing any evident phenotypic changes in the chaperone activity of Hsp90 *per se* (Rutherford and Lindquist, 1998; Bergman and Siegel, 2003). However, this original scenario has recently come under challenge because Hsp90 also acts as a suppressor of retrotransposons and its mutation induces retrotransposon transposition, thus causing a number of secondary mutations (Specchia et al., 2010; Gangaraju et al., 2011).

DNA methylation is commonly observed in a wide range of organisms, from bacteria to plants and animals, although certain model organisms do in fact lack this feature. We propose that changes in DNA methylation in genome regulation systems gave rise to the great diversity of the organisms across the earth. In particular, as mammals developed their particularly specialized DNA methylation system, mammalian evolution was advanced by a series of retrotransposon domestication events. Retrotransposons serve as a double-edged sword in development and evolution, i.e., either harmful or beneficial depending on which time scale is used. The domestication of retrotransposons seems likely to be a very rare event, but once it has taken place, its impact is profound, which is especially the case in mammalian evolution. That may provide the *raison d’etre* for the LTR retrotransposons in the mammalian genome.

## Conflict of Interest Statement

The authors declare that the research was conducted in the absence of any commercial or financial relationships that could be construed as a potential conflict of interest.
